# Epidemiology and characterization of avian infectious bronchitis virus strains circulating in southern China during the period from 2013–2015

**DOI:** 10.1038/s41598-017-06987-2

**Published:** 2017-07-26

**Authors:** Keyu Feng, Feng Wang, Yu Xue, Qingfeng Zhou, Feng Chen, Yingzuo Bi, Qingmei Xie

**Affiliations:** 1 College of Animal Science, South China Agricultural University, Guangzhou, 510642 P.R. China; 2 Key Laboratory of Chicken Genetics, Breeding and Reproduction, Ministry of Agriculture, Guangzhou, 510642 P.R. China; 3 Guangdong Wen’s Foodstuff Group Co. LTD., Guangdong Enterprise Key Laboratory for Animal Health and Environmental Control, Yunfu, 527439 P.R. China; 4 Key Laboratory of Animal Health Aquaculture and Environmental Control, Guangzhou, 510642 P.R. China; 5 South China Collaborative Innovation Center for Poultry Disease Control and Product Safety, Guangzhou, 510640 P.R. China

## Abstract

Two hundred and six strains of avian infectious bronchitis virus (IBV) were isolated from chickens showing signs of disease in southern China during the period from 2013–2015. The nucleotide and amino acid sequences from the isolated field strains were compared to 42 published references. Nucleotide homologies ranged from 63.1–99.9% and amino acid homologies ranging from 60.2–100%. At least seven IBV genotypes were co-circulating in commercial chicken farms in southern China. The IBV isolates were genetically diverse and underwent continuing evolution. The QX-type, TW I-type, and 4/91-type were the most common genotypes during the three-year observation period and accounted for 88.8% of the isolated strains. Notably, the prevalence of the TW I-type strains has been increasing in recent years and has become the most common genotype in China. The emergence of variant IBV strains can be attributed to recombination. Serologic analysis and antigenic 3D cartography of 4 reference and 14 field isolated strains indicated the surveyed IBVs had diverse serology types and that the serotype of the isolated QX-type and TW I-type strains was distinct from the vaccines strains. Therefore, long-term continuing surveillance is necessary for IBV prevention and control.

## Introduction

Avian infectious bronchitis (IB) is an acute and highly contagious disease caused by the avian infectious bronchitis virus (IBV). IB causes substantial economic losses throughout the poultry industry worldwide. IBV affects chickens of all ages causing upper respiratory disease, nephritic syndromes, and decreased egg production^1^. IBV has been circulating in China since 1982. The epidemiology of IBV in China is complicated due to the large geographical area, and multiple IBV genotypes and serotypes are known to coexist^2^. Our previous epidemiology monitoring during 2004 to 2012, found at least seven genotypes of IBV circulating. In that study, the QX-like IBV genotype that causes nephritis had become predominant in China^3–6^. Although IBV vaccines are widely used and most commercial chickens have been vaccinated, there are still frequent outbreaks of IB that cause clinical disease and production problems^7–9^. For example, widespread use of vaccines derived from Massachusetts (Mass) serotypes such as H120 and H52 did not prevent frequent outbreaks of IB in chicken flocks^10, 11^.

IBV belongs to the genus *Gammacoronavirus*, family *Coronaviridae*, and order *Nidovirales*. Its genome is linear, non-segmented, positive-sense, single-stranded RNA that is approximately 27.6 kilobases (kb) in length. As with other coronaviruses, the IBV S1 glycoprotein contains virus neutralizing epitopes and serotype-specific sequences^12, 13^. The S1 protein is involved in infectivity and plays an important role in attachment to the host cell, as well as induction of neutralizing antibodies^14^. Given the importance of the S1 protein virus/host interactions, IBV genetic analysis and research of IBV has been primarily based on the S1 gene^15^.

Variation in the S1 protein is the basis for genotype and serotype classification, defines antigen characteristics, contributes to emergence of IBV variants, and poor vaccine protection^13, 16^. S1 variability is due in part to the lack of proofreading by the RNA-dependent RNA polymerase (RDRP) that introduces mutations into the viral genome during replication. Variation is also introduced by the specific template switching mechanism used by coronaviruses, which frequently leads to genetic recombination^17, 18^. At least 30 serotypes of IBV have been identified worldwide. Vaccine cross-protection between serotypes is minimal^19^. Therefore, phylogenetic and genetic analyses have become tools for monitoring the molecular evolution of IBV and provide a fast and accurate method for genotype classification. In some studies, the genotype analyses have been used to predict IBV serotype and used to inform vaccine selection^20^.

In this study, long-term surveillance was conducted with the aim of identifying IBV strains isolated from commercial chicken farms in south China. During 2013–2015, IBV strains were isolated from flocks with clinical outbreaks. Genetic, phylogenetic, and recombination analyses were performed to study the genetic diversity of the S1 gene in field isolated IBV strains. Cross-neutralization tests and antigenic cartography were performed to determine the serological and antigenic relationship between a part of the isolated and vaccine strains.

## Results

### Clinical characteristics of IBV strains from southern China

**Figure 1:**
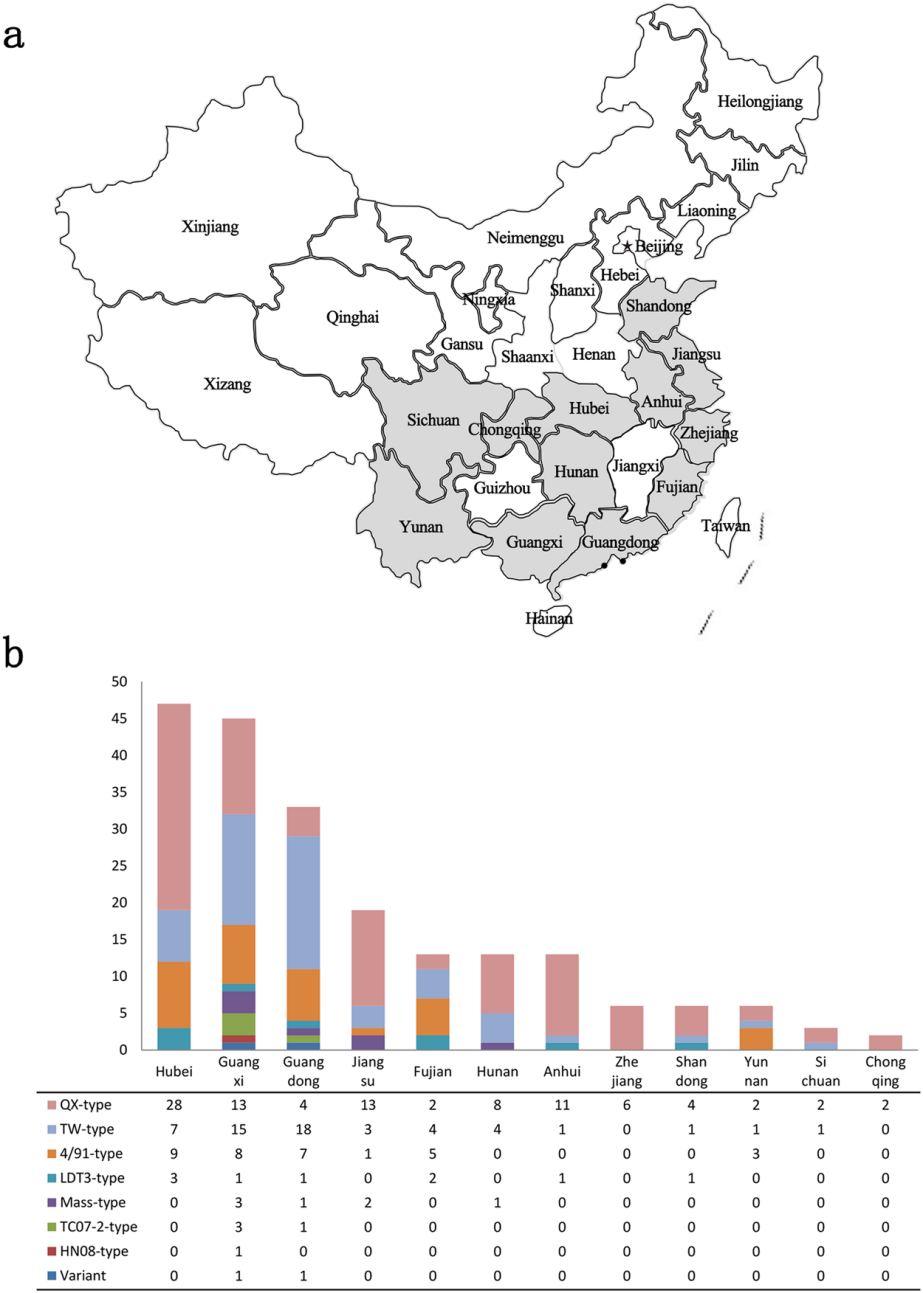
(**a**) The provinces surveyed during the period from 2013–2015 are indicated by shading. The map is created by ArcGIS 9.1 software, http://www.arcgis.com/features. (**b**) The percentage of IBV strains isolated from each province is shown by genotype.

In total, 206 IBV strains were isolated from chicken flocks in southern China during the period from 2013–2015. The collected IBV strains were primarily isolated from flocks of yellow-feathered broilers and broiler breeders. IBV strains were isolated from 12 provinces including Hubei (n = 47), Guangxi (n = 45), Guangdong (n = 33), Jiangsu (n = 19), Fujian (n = 13), Hunan (n = 13) Anhui (n = 13), Zhejiang (n = 6), Shandong (n = 6), Yunnan (n = 6), Sichuan (n = 3), and Chongqing (n = 2) (Fig. 1a). The number of strains collected varied across 2013 (n = 94), 2014 (n = 75), and 2015 (n = 37). Supplementary Table S1 contains the isolation information and main features of each IBV strain isolated during the study.

Clinical IB disease was observed predominately in 10–40 day old chickens (74.7%; 154/206), but IBV was collected from infected chickens ranging from 8–245 days old. Of the 206 collected strains, 153 strains (83.5%) were collected in winter and spring (October to March of the next year), which are cold and wet in southern China. Of the 206 collected strains, 172 strains (83.5%) were associated with typical nephritis symptoms such as swollen specked kidney, distended ureter filled with uric acid, severe dehydration and weight loss. Nephropathogenic IB affected chickens of all ages. Typical respiratory symptoms were observed in 9.7% (20/206) of infected chickens, all of which were less than 40 days-old and included gasping, coughing, sneezing, tracheal rale, and nasal discharge. The prevalence of IB disease in broiler breeders was 6.8% (14/206) and caused a decline in egg production and quality.

### Assessment of the diversity in the S1 gene from IBV field isolates

The S1 gene from each of the field isolates was sequenced and submitted to GenBank (accession numbers KX107628-KX107847). The accession number of each strain is provided Supplementary Table S1. Within the S1 gene, there were eight different nucleotide (1611, 1614, 1617, 1620, 1623, 1626, 1632, and 1635) and deduced amino acid (537, 538, 539, 540, 541, 542, 544, and 545) numbers. The most common (77.2%; 159/206) S1 gene included 1620 nucleotides. Sequence alignment indicated that the other S1 genes contained mutations, insertions and/or deletions resulting in nucleotides differences and different numbers of nucleotides and deduced amino acids. There were eight cleavage site motifs (HRRRR, RRFRR, RRLRR, RRSRR, HRSRR, HRHRR, HRHKR, and HRRKR) among the isolates. The most common cleavage site motifs were HRRRR (51.5%, 106/206), RRFRR (33.5%, 69/206), and RRSRR (9.7%, 20/206). Different from the *Orthomyxovirus* and *Paramyxovirus*, the cleavage site motifs of IBV were not related to host cell, serotype and pathogenicity, but more likely related to geographic origin^21^. However, the cleavage site motifs of IBV has become complex and diverse in many regions with the spread of IBV in worldwide. Similarity analyses showed the similarities of nucleotide and deduced amino acid sequences ranged from 63.1–99.9% and 60.2–100%, respectively. These results indicated that the S1 gene of the field isolates was highly variable. The diversity of S1 gene may attribute to the lack of proofreading by the RDRP and specific template switching mechanism, and it is the mechanism of IBV to evolve and evade the immune surveillance of host.

### Phylogenetic analysis of the IBV genotypes circulating in southern China

**Figure 2:**
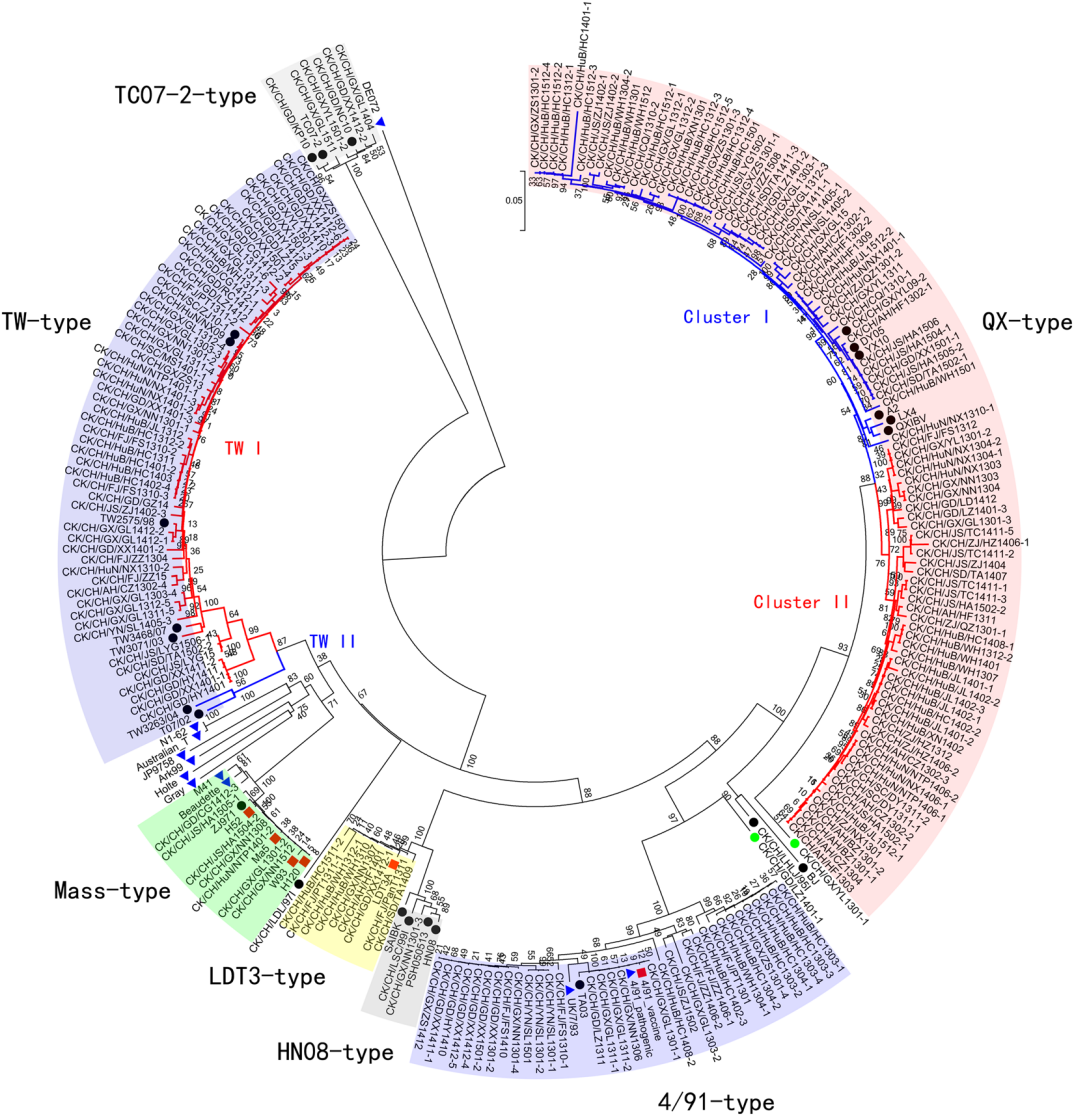
A phylogenetic tree was constructed based on the S1 gene from the 206 isolated field strains and 42 published reference strains using the neighbor-joining method and setting bootstrap 1000 replicates. The vaccine strains that are used in China are marked with ‘’. The Chinese reference strains are marked with ‘’. The foreign reference strains are marked with ‘’. Two variant strains that were the result of recombination were isolated and marked with ‘’. Unmarked strains are field strains isolated in this study.

To assess the genetic relatedness among the IBV strains, a phylogenetic tree was constructed based on the S1 gene nucleotide sequences that contained all 206 field isolates and 42 reference strains. As shown in Fig. 2, there were seven distinct clusters that contained 204/206 field isolates and most of the Chinese reference strains, the QX-type, TW-type, 4/91-type, LDT3-type Mass-type, TC07–2-type, and HN08-type. Two of the field isolates, CK/CH/GX/YL1301-1 and CK/CH/GD/LZ1401-1, were located in two individual evolutionary braches of phylogenetic tree without a reference strain and were considered variants. Three of the Chinese reference strains, BJ, CK/CH/LHLJ/95I, and CK/CH/LDL/97I, clustered into two individual genetic groups without a field isolate. With the exception of the Mass-type and 4/91-type vaccine strains, all the field isolates and Chinese reference strains are evolutionarily distant from the non-Chinese strains (Gray, Holte, Ark99, JP9758, Australian T-strain, N1-62, and DE072) and were located in different evolutionary branches. The proportion of newly isolated field strains from each genotype is shown by province in Fig. 1b, illustrating the complexity and diversity of IBV distribution in southern China.

### Characterization of the QX-type IBV strains circulating in southern China

The QX-type IBV strains included in the study consisted of six reference strains and 95 field strains isolated from all 12 surveillance provinces. The QX-type field stains account for 46.1% (95/206) of isolates. Given the percentage of and geographical distribution of the QX-type viruses, we concluded that this was the predominant genotype of IBV circulating in southern China. The QX-type isolates shared a high degree of nucleotide (91.3%) and amino acid (92%) similarity between strains, and a lower degree of nucleotide (85.7%) and amino acid (85%) similarity with the vaccine strains (H120, H52, Ma5, W93, 4/91, and LDT3-A) used in China.

There were two particularly notable observations regarding the QX-type IBV. First, the percentage of field isolates in the QX-type declined in this surveillance period compared to historical data. From 2004–2012, the QX-type IBV accounted for 50–60% of the total isolates in southern China in our surveillance. In the same period, Other researcher’s study showed that more than 54% of Chinese IBV isolates were QX-type (or LX4-type)^10, 22–24^. During this survey (2013–2015), 46.1% of all field isolates were QX-type, and the prevalence of QX-type IBV was well below average in the provinces of Guangxi (28.9%, 13/45), Guangdong (12.2%, 4/33), and Fujian (15.3%, 2/13). Second, there were two clusters of QX-type IBV, Cluster I and Cluster II (Fig. 2). All the QX-type reference strains in this study including the classical strains QXIBV, A2, and LX4 were grouped in Cluster I. While 48 isolates were grouped to Cluster I, they were in different terminal evolutionary branches from the classical QX-type reference strains (QXIBV, A2 and LX4).

The Cluster II QX-type strains started to emerge in about 2010 (unpublished observation) and they have rapidly increased as a percentage of all QX-type strains. In this study, Cluster II strains account for about 50% (47/95) of the QX-type isolates. Compared to the classical QXIBV strain, the Cluster I strains have 96–98.2% nucleotide sequence similarity and 94.6–96.3% amino acid sequence similarity. The Cluster II strains have slightly lower nucleotide (94.9–97.2%) and amino acid (92–94.3%) similarities to QXIBV. Therefore, all the newly isolated QX-type strains deviated evolutionarily from the classical strains. Sequence alignment of 95 QX-type strains with reference strain QXIBV was performed. The result showed that there were large variations in some nucleotides universally between the two sub-genotype strains (Supplement Table S2). We speculated that the accumulation of nucleotide substitution may contribute to the formation of two sub-genotypes.

### Characterization of the TW I-type IBV strains circulating in southern China

IBV strains in the TW I-type group were the second most common IBV strains identified in southern China. The TW-type originated from Taiwan and contains two subtypes: TW I and TW II. Based on the phylogenetic analyses, all 55 of the strains isolated in this study are members of the TW I-type. The TW-type reference strains utilized here included five Taiwanese strains (TW2575/98, TW3468/07, TW3071/03, TW3263/04, and T07/02) and two strains from mainland China (CK/CH/HuN/NX09 and CK/CH/SC/ZJ10-1). TW I-type strains accounted for 26.7% (55/206) of all isolates and were found in ten provinces: Guangdong, Guangxi, Hubei, Fujian, Hunan, Jiangsu, Anhui, Shandong, Yunnan, and Sichuan. The TW I-type strains were more prevalent than the QX-type viruses in Guangdong (33.3% vs 12.2%) and Guangxi (54.5% vs 28.9%). The TW I-type isolates shared 95.9–99.5% and 94.5–98.8 nucleotide and amino acid identity, respectively, with the Taiwanese reference strain TW2575/98, but less than 82% and 82.3% nucleotide and amino acid identity, respectively, with the strains from other mainland China and vaccine strains (H120, H52, Ma5, W93, 4/91, and LDT3-A). These observations may suggest the different serotype and low protection between vaccine strains and TW I-type isolate strains.

### Characterization of the 4/91-type IBV strains circulating in southern China

IBV strains in the 4/91-type group were the third most common IBV strains identified in southern China. The strains that were classified as the 4/91-type consisted of 33 new isolates and four reference strains. The field isolates were isolated from Hubei, Guangxi, Guangdong, Fujian, Jiangsu, and Yunnan. The reference strains were 7/93, TA03, and the pathogenic and vaccine 4/91 strains. The newly isolated 4/91-type strains accounted for 16.0% (33/206) of all the field isolates. Compared to each other, the 4/91-type IBV isolates shared greater than 94.1% and 93% nucleotide and amino acid identity, respectively. Compared to the vaccine 4/91 strain, they had greater than 96% and 94.6% nucleotide and amino acid identity, respectively. There were five field isolates (CK/CH/GX/GL1301-1, CK/CH/GX/NN1306, CK/CH/GX/GL1311-1, CK/CH/GX/GL1311-2, and CK/CH/GD/LZ1311) that clustered and shared greater than 99% homologous at the nucleotide and amino acid levels with the pathogenic and vaccine 4/91 strains. The S1 nucleotide sequences were compared between the 5 field isolates and reference 4/91 strains to determine whether the field isolates originate from pathogenic (Supplement Fig. S1). The five field isolates and the pathogenic 4/91 strain has the same nucleotides in positions 283, 1522, and 1589. In contrast, the vaccine 4/91 strain has mutations at these three positions that lead to attenuating amino acid substitutions (95, 508 and 530)^25^. The membrane (M) and nucleocapsid (N) genes from the field isolates located in phylogenetic clusters and shared high nucleotide similarity with Chinese reference strains rather than 4/91 vaccine or pathogenic strain (Supplement Fig. S2). These results indicated, the five field isolates were not likely originated from vaccine strain, but from the pathogenic 4/91 strain and that there may have been recombination between the pathogenic 4/91 strain and other circulating IBV strains.

### Description of the less common IBV strains circulating in southern China

Combined, the remaining four genotypes accounted for approximately 10% of the field isolates. Fewer than 10 isolates each were found of the LDT3-type (n = 9), Mass-type (n = 7), TC07-2-type (n = 4), and HN08-type (n = 1). The LDT3-type vaccine strain LDT3-A and Mass-type vaccine strains (H120, H52, Ma5 and W93) are widely used in China, and the field isolates of the two genotypes were closely related to and shared more than 97.3% nucleotide similarity of S1 gene with vaccine strains of same genotype. Similar to the 4/91-type, the M or N genes of isolated strains also had relatively distant evolutionary distance and low similarity with the corresponding vaccine strain (Supplement Fig. S2). This suggested that the vaccine strains may have recombined with other circulating IBV strains. The TC07-2-type isolates were evolutionary distant from the other genotypes and had low nucleotide sequence similarity (63.1–68.1%) with vaccine and other genotype strains. The TC07-2-type stains were all isolated in Guangdong and Guangxi. One field isolate (CK/CH/GX/NN1301-3) from Guangxi was an HN08-type IBV. It had less than 85.6% and 84.8% nucleotide and amino acid identity, respectively with the vaccine strains.

### Description of the recombinant IBV strains circulating in southern China

**Figure 3:**
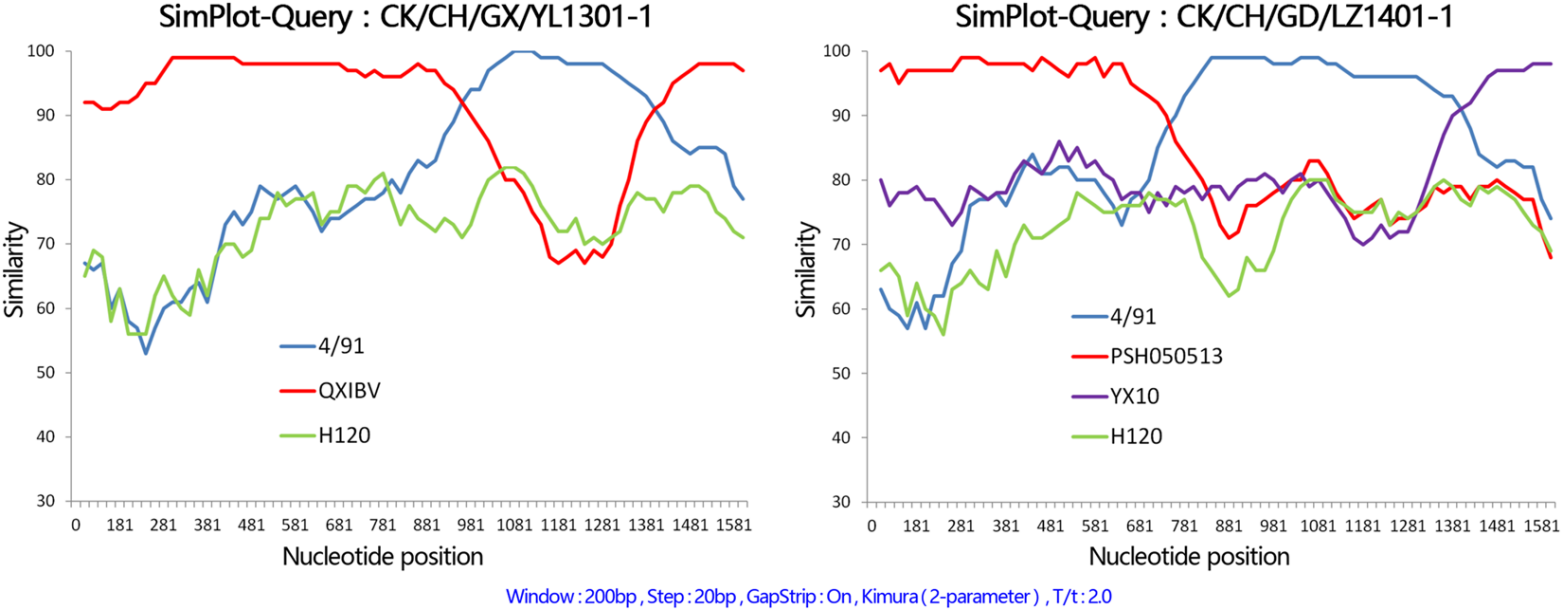
SimPlot analysis of variant strains CK/CH/GX/YL1301-1 and CK/CH/GD/LZ1401-1. The S1 gene from CK/CH/GX/YL1301-1 was a recombinant of the reference strains QXIBV and 4/91. The S1 gene from CK/CH/GD/LZ1401-1 was a recombinant of the reference strains PSH050513, 4/91, and YX10 after two recombination events. H120 was used as the outlier sequence. The y-axis gives the percent identity within a sliding window 200 bp wide centred on the position plotted, with a step size between plots of 20 bp.

Two of the field isolates clustered to two individual genogroups that did not include reference strains based on their S1 genes and termed variants. The variant CK/CH/GX/YL1301-1 isolated from Guangxi shared less than 89.6% and 88.2% nucleotide and amino acid similarity with strains from other genotypes. A Recombination Detection Program (RDP) analysis showed that this variant was derived from recombination between QXIBV (major parent) and 4/91 (minor parent; Table 1). Recombination occurred between nucleotides 919–1300. This region (919–1300) was 99.3% similar to 4/91, while the rest of the S1 gene was 97.3% similar to QXIBV. The variant CK/CH/GD/LZ1401–1 was isolated from Guangdong and shared less than 89.9% and 87.5% nucleotide and amino acid similarity with strains from other genotypes. The RDP analysis showed that this variant was formed from regions of three reference strains and required two recombination events. From nucleotide 1–735 the S1 gene of CK/CH/GD/LZ1401-1 was 98.2% similar to the HN08-type strain PSH050513, from nucleotide 736–1391 it was 97.8% similar to the 4/91 strain and the remainder of S1 was 98.6% similar to the QX-type strain YX10. The results of RDP analysis were confirmed by a SimPlot analysis (Fig. 3). These results suggest that recombination between strains of differnet genotypes may contribute to the emergence of novel IBV variants.

**Table 1:** S1 gene recombination in variant IBV strains.

Variant Strain	Breakpoint Positions		Minor Parent^a^			Major Parent^b^			p-value^c^
	Begin	End	Strain	Genotype	Similarity	Strain	Genotype	Similarity	
CK/CH/GX/YL1301-1	919	1300	4/91	4/91-type	99.30%	QXIBV	QX-type	97.30%	9.83E-39
CK/CH/GD/LZ1401-1	736	1391	4/91	4/91-typ	97.8%	PSH050513	HN08-type	98.2%	1.78E-25
	1401	1623	YX10	QX-type	98%	PSH050513	HN08-type	98.2%	2.10E-21

^a^Minor parent = Sequence closely related to the transferred fragment in the recombinant.

^b^Major parent = Sequence most closely related to the sequence surrounding the transferred fragment.

^c^p-value of RDP method.

### Cross-neutralization assay results for 4 reference strains and 14 field isolates

The live attenuated vaccine strains used in China include Mass-type strains (H120, H52, 28/86, Ma5, W93), 4/91, LDT-3A and so on. To evaluate Cross-neutralization reactivity between the vaccine and circulating strains, the different genotype vaccine strains widely used in China (H120, 4/91, LDT3-A), QX-type attenuated YX10-D90 and 14 of the field isolated strains was selected to prepare serum. The ability of each immune serum to neutralize infection with the IBV strains was tested in specific pathogen-free (SPF) chicken embryos.

In general, the highest titers of neutralizing antibodies were found against homologous strains, while the titer of neutralizing antibodies against heterologous strains was more varied (Table 2). Two-way cross-neutralizing activity was not detectable in some cases. For example, the 4/91 antiserum did not neutralize CK/CH/HuB/HC1501 virus, and the CK/CH/HuB/HC1501 antiserum did not prevent infection with 4/91 virus. One-direction neutralization was also observed. For example, the 4/91 antiserum prevented infection with CK/CH/GX/NN1301-3, but the CK/CH/GX/NN1301-3 antiserum did not prevent infection with 4/91. There was little cross-neutralizing activity (dilution endpoint less than 64) between 3 vaccine strains and the vaccines also showed low level neutralizing activity against most of the field isolates, particularly vaccine H120, did not elicit effective neutralization against the QX-type and TW I-type IBV isolated strains.

**Table 2:** Neutralization activity of immune sera against each IBV strain.

Genotype	IBV strain	Antiserum																	
		1	2	3	4	5	6	7	8	9	**10**	11	12	**13**	**14**	15	16	17	18
QX-type	1. YX10-D90^a^	628 ^c^	284	368	256	564	312	8	4	12	32	16	8	-^d^	64	64	354	92	—
	2. CK/CH/AH/BZ1301-1	512	386	326	256	256	64	8	4	8	64	64	46	—	64	94	286	84	128
	3. CK/CH/CQ/1310-2	416	247	476	364	446	132	4	—	—	32	28	18	8	16	128	32	64	32
	4. CK/CH/SD/TA1407	446	256	256	512	256	256	—	—	26	46	4	32	—	16	64	437	98	84
	5. CK/CH/GD/LD1412	512	301	128	512	624	128	—	4	36	16	4	12	4	32	32	128	64	32
	6. CK/CH/HuB/HC1501	21	71	64	64	32	386	4	—	8	256	324	256	—	—	32	32	196	128
TW I-type	7. CK/CH/GX/ZS13	—	—	8	—	16	—	324	481	473	32	—	—	4	32	—	—	32	8
	8. CK/CH/GD/GZ14	4	—	8	—	—	—	288	512	476	—	32	8	—	8	8	—	—	—
	9. CK/CH/FJ/ZZ15	4	32	16	—	—	—	292	314	526	84	—	—	8	32	—	4	32	8
4/91-type	**10. 4/91 vaccine** ^b^	23	13	8	32	64	256	16	—	32	512	363	256	12	64	—	32	64	16
	11. CK/CH/FJ/PT1301	36	21	8	26	64	322	8	—	—	452	446	328	—	16	64	64	112	8
	12. CK/CH/GX/ZS1412	4	—	—	18	54	294	—	—	—	416	314	476	8	32	64	8	64	32
Mass-type	**13. H120 vaccine**	—	4	—	—	—	4	—	—	—	32	64	128	480	16	8	64	16	—
LDT3-type	**14. LDT3-A vaccine**	8	12	26	12	—	—	4	32	64	18	8	64	16	512	128	64	—	32
HN08-type	15. CK/CH/GX/NN1301-3	4	18	32	16	16	—	—	32	32	256	—	8	32	476	256	—	—	16
TC07-2-type	16. CK/CH/GX/GL1404	446	386	256	256	64	4	—	4	16	—	4	12	64	64	—	512	—	—
Variant	17. CK/CH/GX/YL1301-1	64	48	—	12	—	16	4	4	—	64	18	32	—	—	32	—	476	84
	18. CK/CH/GD/LZ1401-1	386	32	18	—	4	—	—	—	4	32	4	8	18	8	32	4	64	256

^a^YX10-D90 is an attenuated IBV strain that is being considered as a candidate for a QX-type vaccine.

^b^Major commercial IBV vaccine strains used in China (H120, LDT3-A, and 4/91) are shown in boldface.

^c^The reciprocal of the dilution endpoint is shown.

^d^Neutralization activity was not detected.

### Serological analysis and 3D antigenic cartography of 4 reference strains and 14 field isolates

Based on the results of the cross-neutralization assay, the antigenic relatedness values (ARV) were calculated for each of the tested IBV strains (Table 3). The ARV between two strains lower than 25% indicated different serotypes^24, 26^.

**Table 3:** Antigenic relatedness values (%) among the 4 reference and 14 field isolates of IBV.

IBV strain	Strain																	
	1	2	3	4	5	6	7	8	9	10	11	12	13	14	15	16	17	18
1. YX10-D90	100	**77** ^a^	**72**	**60**	**86**	16	0	<5	<5	<5	<5	<5	0	6	<5	**70**	14	0
2. CK/CH/AH/BZ1301-1		100	**71**	**57**	**56**	17	0	0	<5	7	9	0	0	6	13	**87**	15	8
3. CK/CH/CQ/1310-2			100	**62**	**44**	21	<5	0	0	<5	<5	0	0	<5	18	18	0	<5
4. CK/CH/SD/TA1407				100	**64**	23	0	0	0	8	<5	<5	0	<5	9	**65**	7	0
5. CK/CH/GD/LD1412					100	13	<5	0	0	6	<5	<5	0	0	6	16	0	<5
6. CK/CH/HuB/HC1501						100	0	0	0	**58**	**51**	**64**	0	0	0	0	12	0
7. CK/CH/GX/ZS13							100	**91**	**90**	6	0	0	<5	<5	0	0	<5	0
8. CK/CH/GD/GZ14								100	**74**	0	0	0	0	<5	<5	0	0	0
9. CK/CH/FJ/ZZ15									100	10	0	0	0	9	0	<5	0	<5
10. 4/91 vaccine										100	**83**	**66**	14	7	0	0	13	6
11. CK/CH/FJ/PT1301											100	**70**	0	<5	0	<5	10	<5
12. CK/CH/GX/ZS1412												100	<5	6	6	<5	10	<5
13. H120 vaccine													100	<5	<5	13	0	0
14. LDT3-A vaccine														100	**68**	11	0	6
15. CK/CH/GX/NN1301-3															100	0	0	9
16. CK/CH/GX/GL1404																100	0	0
17. CK/CH/GX/YL1301-1																	100	21
18. CK/CH/GD/LZ1401-1																		100

^a^Antigenic relatedness values (ARV) ≥ 25 are shown in boldface indicating that the pair of strains belong to the same serotype.

**Figure 4:**
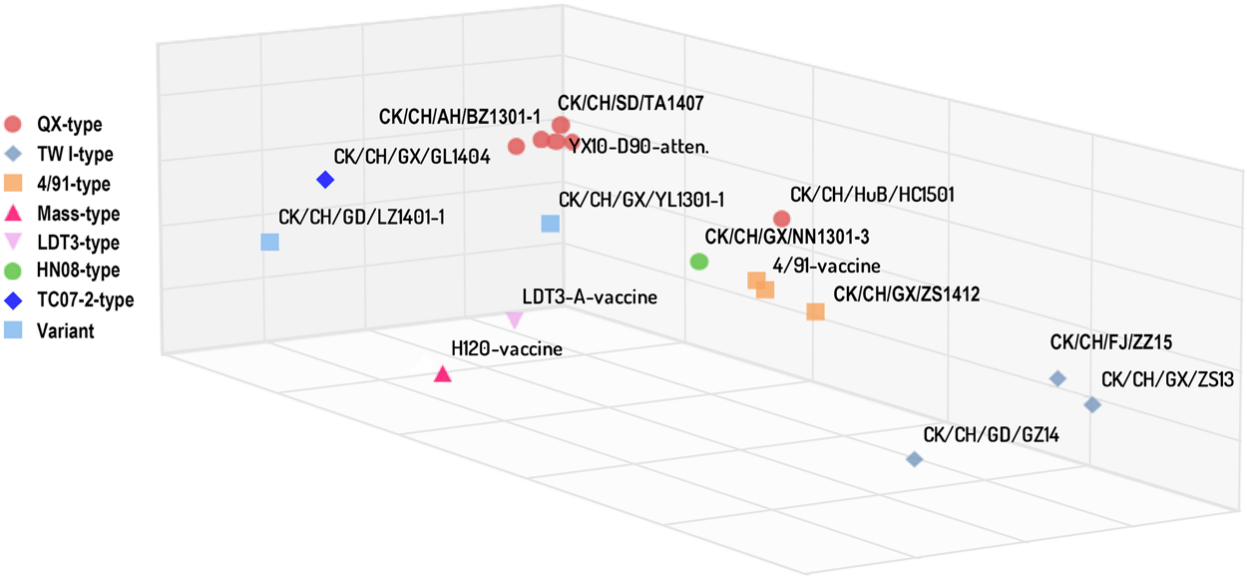
The antigenic 3D cartography for 3 vaccine strains, the attenuated YX10-D90 strain, and 14 field isolated IBV strains. Viruses of the same genotypes are labelled with same tag. One unit in the 3D map corresponds to a two-fold change in the cross-neutralization assay. Distances between two viruses greater than two units on the 3D antigenic cartography map were considered different serotype strains.

Of the 18 IBVs tested, 16 grouped into one of five serotypes and two (variant IBVs) had unknown serotypes. The vaccine strains H120, 4/91 and LDT3-A belong to distinct serotypes (ARV < 15%). The four QX-type isolated strains, attenuated YX10-D90, and the TC07-2-type strain CK/CH/GX/GL1404 shared the same serotype (44% < ARV < 86%) and differed from the serotypes of vaccine strains (H120, 4/91 and LDT3-A). Surprisingly, the QX-type CK/CH/HuB/HC1501 isolated did not have the same serotype as the other QX-type strains, and shared a serotype with the 4/91-type strains (51% < ARV < 64%). The three TW I-type strains had a distinct serotype from other genotypes and 3 vaccine strains (ARV < 5%). The HN08-type CK/CH/GX/NN1301-3 strain and the LDT3-A vaccine had the same serotype (ARV = 68%). The two variant strains CK/CH/GX/YL1301-1 and CK/CH/GD/LZ1401-1 were not antigenically similar and did not share serotypes with their corresponding parent strains (QX-type and 4/91-type, HN08-type, 4/91-type and QX-type). 3D antigen mapping is shown in Fig. 4, a distance greater than 2 units between types is considered as different serotypes^27^. The 3D mapping results were consistent with the ARV analysis.

We then comparing the concordance between classification based on serotype and genotype. The 3 tested vaccine strains showed concordance between phylogenetic and serological analysis, They belong to three independent genotypes and three serotypes, and distinct from the predominant field isolates. Of the 14 tested field isolates, nine strains demonstrated concordance between genotype and serotype, three strains were not concordant, and two variant strains had an unknown serotype inconsistent with that parental genotype.

## Discussion

This study is part of a long-term surveillance program to understand the epidemiology of IBV and to characterize the circulating IBV strains found on commercial chicken farms in south China. Given the genomic and antigenic variability of IBV and poor vaccine protection, characterizing emergent IBV strains based on genotype and serotype is critical to preventing IB disease. Typically serotype is determined based on cross-neutralization assays. However, these assays are limited for IBV by the lack of a standard positive serum. Therefore, genotype classification based on the S1 gene has become the primary method of classifying IBV strains.

Although different genotype classifications and denomination of Chinese IBV have been proposed^22, 23^, the classification of major genotypes such as QX-type (A2-type or LX4-type), TW-type, 4/91-type (793/B-type or CR88-type) and Mass-type was basically coincident. For purposes of this study, the genotype classification reported by Luo *et al*. and reference strains reported by other researchers^4, 10, 22, 24^ have been used.

During the period from 2013-2015 there were 7 distinct genotypes of IBV (based on the S1 gene) circulating in southern China. Of these, 89.8% represented the QX-type, TW I-type, or 4/91-type IBV. The QX-type (also called LX4-type or A2-type) was the most common (46.7%) and was distributed in all surveyed provinces. QXIBV was first recognized in 1996 and caused proventriculitis. In contrast, the QX-type IBVs prevalent in China today primarily cause typical nephritis. Since the discovery of QXIBV, LX4, and A2 strains in 1990s, the QX-type has become the predominant IBV genotype in China and spread to Europe^28–30^. Previously, the QX-type IBV accounted for 50–60% of field IBV isolates from 2004–2012^3–6^. Here, we saw a slight decrease in the prevalence of QX-type IBV. One hypothesis is that the development and use of a QX-type attenuated vaccine has reduced the level of infection in some areas^31–33^. The two clusters of S1 genes for the QX-type strains collected here were variable and evolutionarily distinct from the classical strains (QXIBV, LX4, and A2). This may be attributable to the accumulation of mutations, including insertions and deletions, and recombination of small segments of the S1 gene. Additional studies are needed to determine whether these genetic changes influence the antigenic characterization of QX-type strains.

The TW-type strains were firstly isolated from Taiwan. Due to the geographical separation between Taiwan and mainland China, the IBV strains found in Taiwan are evolutionarily distinct from IBV genotypes in mainland China and other countries^34^. The TW-type is divided into the TW I and TW II subgroups. The strains isolated from Taiwan before 1990 were primarily TW II genotypes associated with respiratory symptoms. The TW I genotype strains isolated after the 1990s were primarily associated nephritis. Vaccination with Mass-type (H120, H52, and Ma5) and 4/91 vaccines is common in Taiwan, but TW I-type IBV continues to break out frequently^35^. Prior to 2009, the TW-type strain was rare in mainland China^5, 23^. However, since 2009 the prevalence of TW I-type IBV has been increasing in mainland China from 7.5% (2009–2010) to 16.1% (2011–2012), and TW II-type isolates are also being found^6^. In this study, the TW I-type strains accounted 27.4% of isolated strains. More isolates of this genotype had been reported in China mainland^36, 37^. Notably, the prevalence of TW I-type strains was greater than QX-type strains in Guangdong Guangxi, and Fujian, and was the predominant genotype in all three provinces. Geographically, these three provinces are all on the southeast coast of China and close to Taiwan. It is possible that migrating birds introduced the TW-type strains to these areas. The TW I-type strain might be increasing in prevalence due to selective pressure from vaccination on other genotypes and the lack of vaccine efficacy against the TW I-type strains. Currently, there is little known about the biological characteristics of TW I-type IBV in mainland China.

The 4/91 (also called 793/B-type or CR88-type) vaccine and pathogenic strains originated from Europe. The prevalence of 4/91 strains in China is controversial. Here, the 4/91-type strains accounted for 16.4% of strains isolates from 2013–2015. We found evidence that the pathogenic 4/91 strain is actively involved in recombination with native Chinese strains, suggesting that 4/91 has become an important gene donor in the genetic evolution of IBV in China. While the 4/91 vaccine is not officially approved for use in China, it is widely used on chicken farms. There is a risk that the recombination between the 4/91 vaccine strains and other virulent native Chinese strains (such as QX-type) and formed recombinant virulent strain. Therefore, it is necessary to continue monitoring the prevalence and evolution of the 4/91-type IBV.

The remaining IBV genotypes (LDT3-type, Mass-type, HN08-type, and TC07–2-type) were rate and combined accounted for only 10.2% of isolates. The LDT3-type and Mass-type strains include common vaccine strains (LDT3-A, H120, H52, W93, and Ma5). The Mass-type vaccine strains are used worldwide. The LDT3-A vaccine strain used in China was derived from an attenuated ck/CH/LDT3 IBV strain isolated in Guangdong province in 2003. These vaccine strains are approved for use in China and widely used. Vaccination reduces the incidence of IB disease, but also increases the risk of recombination with field strains. The HN08-type strains originated from multiple recombination events and accounted for 25% of the IBV strains isolated in Chinese during 2008–2009^4^. Only one HN08-type strain was isolated in this study. The TC07–2-type IBV was first isolated in Jiangsu province in 2007 (TC07-2 strain) and is evolutionarily distant from other genotypes. The origin and characteristics of TC07-2-type IBV are still unknown.

Two variants with different parental genotypes were detected by recombination analysis in this study. Recombination is an important mechanism of IBV evolution that generates field variants^38^. The unique discontinuous transcription system and the viral polymerase “jumping” may contribute to the high RNA recombination frequency in IBVs. A recombination event can occur when the viral polymerase switches from one template to another during genomic synthesis in a host that is infected by two or more strains of IBV^11^. Recombination reportedly took place between the Chinese QXIBV and classical IBV strains in countries around China, such Thailand and Korea, producing new variant strains and subgroups^39–41^. Thus, recombination continues to be an important source of emergent IBV variant strains.

There are at least 30 known serotypes of IBV, which is one of the challenges to IBV prevention and control. In this study, serotype classification was determined based on a two-way cross-neutralization assay performed in SPF chicken embryos. Relevant for vaccination control, the viral genotypes and serotypes of the vaccine strains were distinct from those of the most prevalent Chinese strains. We also observed a marked inconsistency between the viral genotype and serotype. This may be attributable to mutation of antigenic epitopes in S1 or immunity elicited to other IBV proteins such as S2 or N. Identifying the antigenic profile of emergent IBV isolates is important for vaccine development and effective control of disease outbreaks caused by variant strains. Antigenic cartography has been widely used to understand antigenic variation of the influenza A virus^27^. In this study, 3D antigenic cartography was applied to IBV. The results of the 3D cartography analysis were consistent with serotype classification by ARV. 3D antigenic cartography has the advantage of being intuitive and can be more easily visualized. Therefore, this may be a useful technique for identifying antigenic variants and vaccine candidates for rapidly evolving variants of IBV.

## Materials and Methods

### Vaccine strains

H120 (Dahuanong Animal Health Products Co., Ltd., Guangdong, China Lot: 20140312), LDT3-A (Weike Biotechnology Development Co., Ltd., Harbin, China Lot: 20131202), 4/91 (INTERVET International B. V. Boxmeer, Holland Lot: 20110921), Attenuated YX10-D90 was preserved in the authors’ laboratory.

### Virus isolation

The study was conducted in southern China during the period from 2013–2015. Samples were obtained from the kidney, trachea, and lung of broiler chickens and broiler breeder flocks suspected of being IBV infected. Typical clinical signs of diseased birds included respiratory symptoms, nephritis, and decreased egg production. The samples were frozen and thawed three times, suspended in phosphate-buffered saline (PBS) contained 200 U/mL penicillin and 200 µg/mL streptomycin, and then centrifuged at 7,000 × *g* for 5 min. After storage at 4 °C for 12 h, the supernatant was inoculated into the allantoic cavity of 9-day-old SPF chicken embryos. One to five blind passages were performed until characteristic embryo changes, such as dwarfing, stunting, or curling of the embryos were observed^46^. RNA was extracted from the allantoic fluids using the AxyPrep^TM^ Body Fluid Viral DNA/RNA Miniprep Kit (Axygen, Hangzhou, China) per the manufacturer’s instructions. IBV infection was verified by reverse transcription-polymerase chain reaction (RT-PCR) for the M gene (MF: 5′-CCTAAGAACGGTTGGAAT-3′, MR: 5′-TACTCTCTACACACACA C-3′). The virus isolation and follow-up experiments were carried out in biosafety level 2 (BSL-2) biocontainment laboratory.

### RT-PCR and sequencing of the S1 gene

A pair of S1 specific primers (S1-F: 5′-AAGACTGAACAAAAGACCGACT-3′, S1-R: 5′-CAAAACCTGCCATAACTAACAT A-3′) was designed using the Primer Premier 5.0 software. RT-PCR was preformed using the PrimeScript One Step RT-PCR Kit Ver. 2 (Takara, Dalian, China). The RT-PCR products were analyzed by electrophoresis on a 1.0% agarose gel and then observed using an ultraviolet transilluminator. Each RT-PCR product was ligated into the pMD19-T vector (TaKaRa) and transformed into DH5α *Escherichia coli* competent cells. Positive clones were sequenced by Shanghai Sang-gong Biological Engineering Technology & Services Co., Ltd (Shanghai, China).

### Genetic and phylogenetic analysis of the S1 gene

The nucleotide and deduced amino acid sequences of the S1 genes from all the IBV isolates were aligned using the Editseq program and analyzed for homology with 43 reference IBV strains published in GenBank using the MegAlign program. Both programs are available in the Lasergene package (DNASTAR, Madison, WI, USA). The backgrounds and accession numbers of the reference strains are listed in supplemental data. Phylogenetic analysis of the nucleotide sequences and deduced amino acid sequences of the S1 gene was performed with the neighbor-joining method using MEGA version 7.0. The bootstrap values were determined from 1000 replicates of the original data.

### Recombination analysis of the S1 gene of variant IBV strains

The S1 gene of variant IBV strains and their parental strains were identified with RDP version 4.7 and Simplot version 3.5.1. Reference strains were used as hypothetical parental strains. Methods of detecting recombination included: RDP, GENECONV, Bootscan, Maxchi, Chimaera, sisscan, phylpro, LARD, and 3Seq. The default parameters were used for each of the analyses.

### Cross-neutralization assays

Immune sera were produced against the vaccine strains H120, Ma5, 4/91, and LDT3-A, the attenuated strain YX10-D90, and 14 of the field isolated strains. The immune sera were prepared as described by Gao *et al*.^9^. To determine the titer of neutralizing antibody in the immune sera, each serum sample was serially diluted two-fold with sterile PBS and mixed with 200 EID50 of each IBV strain. After incubation for 1 h at 37 °C, the virus-serum mixtures were inoculated into the allantoic cavity of SPF chicken embryos and incubated for 7 days. The end-point titer in each serum sample was calculated using the Reed and Muench method^42^. ARVs were calculated using the formula published by Archetti and Horsfall^43^. ARV lower than 25% were considered independent serotypes^24, 26^. The 3D antigenic cartography was constructed using an online antigenic cartography resource AntigenMap 3D (http://sysbio.cvm.msstate.edu/software/AntigenMap/)^27^.

### IBV reference strains published in GenBank

Forty two IBV strains published in GenBank were selected for the phylogenetic and alignment analysis. The backgrounds and accession numbers of the reference strains are listed in Supplement Table S3.

### Ethics statement

Institutional and national guidelines for the use and care of laboratory animals were closely followed. The use of animals in this study was approved by the South China Agricultural University Committee for Animal Experiments (approval ID: 201304152).

## Electronic supplementary material

Supplementary Information
