# Supplementary material for: Epidemiology and characterization of avian infectious bronchitis virus strains circulating in southern China during the period from 2013–2015
Source: Sci Rep. 2017 Jul 26;7:6576. doi: 10.1038/s41598-017-06987-2 (PMC5529424; doi:10.1038/s41598-017-06987-2)
Supplement: Supplementary file 1 — Supplementary Information [file 41598_2017_6987_MOESM1_ESM.pdf]

Epidemiology and characterization of avian infectious bronchitis virus strains circulating in southern China during the period from 2013–2015

Keyu Feng, Feng Wang, Yu Xue, Qingfeng Zhou, Feng Chen, Yingzuo Bi, Qingmei Xie*

	280	1520	1590
4/91 vaccine	TCAGTTGCACAG	AATTTAATTGGC	AAACCCACTAAC
4/91 pathogenic	......T.....	......G.....	....T.......
CK/CH/GX/GL1301-1	......T.....	......G.....	....T.......
CK/CH/GX/NN1306	......T.....	......G.....	....T.......
CK/CH/GX/GL1311-1	......T.....	......G.....	....T.......
CK/CH/GX/GL1311-2	......T.....	......G.....	....T.......
CK/CH/GD/LZ1311	......T.....	......G.....	....T.......

Supplement Fig. S1 Sequence alignment of S1 genes from the CK/CH/GX/GL1301-1, CK/CH/GX/NN1306, CK/CH/GX/GL1311-1, CK/CH/GX/GL1311-2, CK/CH/GD/LZ1311, and the 4/91 vaccine and pathogenic strains. The five field isolated strains have the same nucleotide as the pathogenic 4/91 strain at positions 283, 1522, and 1589. In contrast the 4/91 vaccine strain is mutated at those three positions. The nucleotide positions are given from the ATG that encodes the start codon of S1.

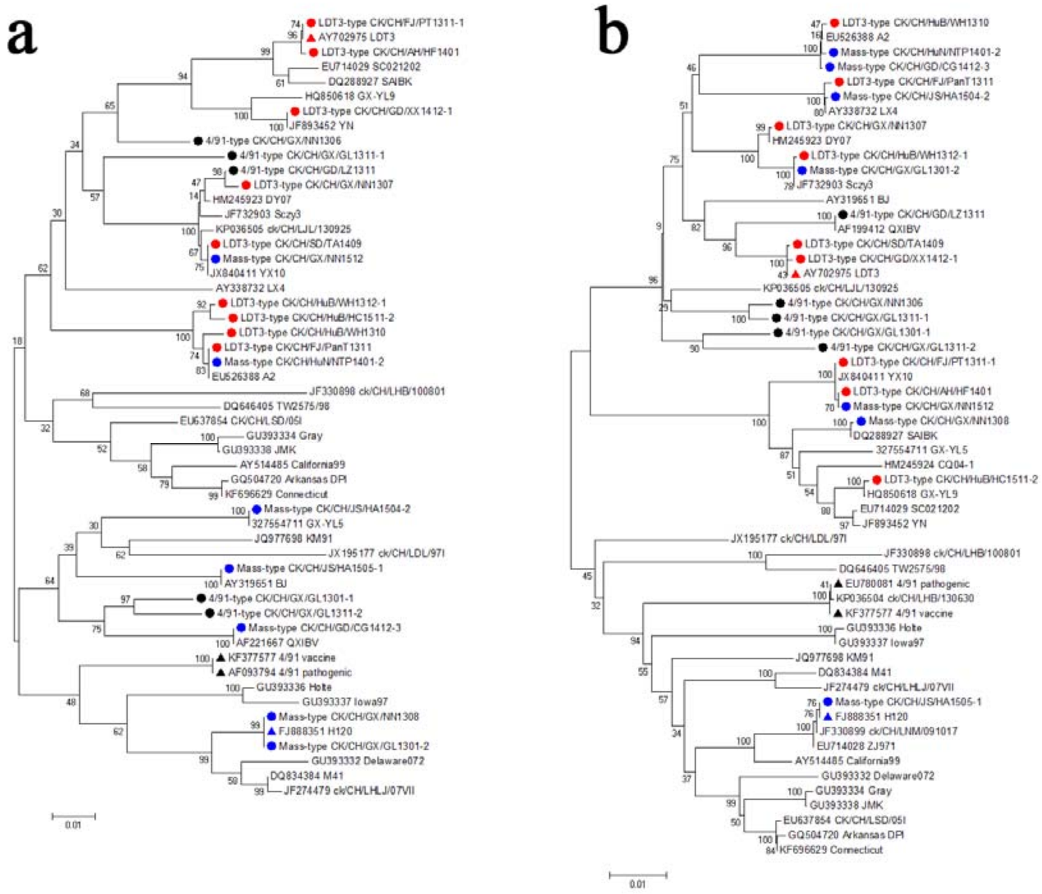

Supplement Fig. S2 Phylogenetic trees were constructed based on the M gene (a) and N gene (b) from the five 4/91-type, nine LDT3-type，seven Mass-type isolated field strains and published reference strains using the neighbor-joining method and setting bootstrap 1000 replicates. The M gene and N gene of five 4/91-type isolated field strains (marked with '●') located in different clusters with 4/91 vaccine or pathogenic strains (marked with '▲'). Similar to the 4/91-type, the M or N genes of nine LDT3-type (●) and seven Mass-type (●) isolated strains had relatively distant evolutionary distance and low similarity with the corresponding vaccine strains (marked with '▲' and '▲') in M gene or N gene.

Supplement Table S1 Background and S1 gene information of the IBV isolated strains in south China during 2013-2015

IBV iolates	Province[a]	The time of isolation	Production type	days of age	Pathogenicity type	Length of S1 gene (nt/aa)[b]	Cleavage recognition motifs[c]	Genotype	Accession Number
CK/CH/AH/BZ1301-1	Anhui	Jan.2013	broiler	28	Nephropathogenic	1620nt/540aa	HRRRR[d]	QX-type	KX107628
CK/CH/AH/BZ1301-2	Anhui	Jan.2013	broiler	49	Nephropathogenic	1620nt/540aa	HRRRR	QX-type	KX107629
CK/CH/FJ/PT1301	Fujian	Jan.2013	broiler	27	Nephropathogenic	1617nt/539aa	HRRRR	4/91-type	KX107649
CK/CH/GD/XX1301-2	Guangdong	Jan.2013	broiler	45	Nephropathogenic	1620nt/540aa	RRSRR	4/91-type	KX107675
CK/CH/GX/GL1301-2	Guangxi	Jan.2013	broiler	14	Respiratory	1611nt/537aa	RRFRR	Mass-type	KX107693
CK/CH/GX/GL1301-3	Guangxi	Jan.2013	broiler	17	Nephropathogenic	1620nt/540aa	HRRRR	QX-type	KX107694
CK/CH/GX/GL1301-1	Guangxi	Jan.2013	broiler	67	Nephropathogenic	1617nt/539aa	RRSRR	4/91-type	KX107692
CK/CH/GX/NN1301-2	Guangxi	Jan.2013	broiler	10	Nephropathogenic	1620nt/540aa	RRFRR	TW I-type	KX107717
CK/CH/GX/NN1301-1	Guangxi	Jan.2013	broiler	16	Nephropathogenic	1620nt/540aa	RRFRR	TW I-type	KX107716
CK/CH/GX/NN1301-4	Guangxi	Jan.2013	broiler	17	Nephropathogenic	1620nt/540aa	RRSRR	4/91-type	KX107719
CK/CH/GX/NN1301-3	Guangxi	Jan.2013	broiler breeder	78	Nephropathogenic	1632nt/544aa	RRFRR	HN08-type	KX107718
CK/CH/GX/YL1301-1	Guangxi	Jan.2013	broiler	18	Respiratory	1620nt/540aa	HRRRR	Variant	KX107728
CK/CH/GX/YL1301-2	Guangxi	Jan.2013	broiler	18	Nephropathogenic	1620nt/540aa	HRRRR	QX-type	KX107729
CK/CH/GX/ZS1301-3	Guangxi	Jan.2013	broiler	22	Respiratory	1617nt/539aa	HRRRR	QX-type	KX107736
CK/CH/GX/ZS1301-4	Guangxi	Jan.2013	broiler	18	Nephropathogenic	1617nt/539aa	HRSRR	4/91-type	KX107737
CK/CH/GX/ZS1301-1	Guangxi	Jan.2013	broiler	19	Nephropathogenic	1617nt/539aa	HRRRR	QX-type	KX107734
CK/CH/GX/ZS1301-2	Guangxi	Jan.2013	broiler	22	Nephropathogenic	1620nt/540aa	HRRRR	QX-type	KX107735
CK/CH/HuB/WH1301	Hubei	Jan.2013	broiler	33	Nephropathogenic	1620nt/540aa	HRRRR	QX-type	KX107777
CK/CH/HuB/XN1301	Hubei	Jan.2013	broiler	18	Respiratory	1617nt/539aa	HRRRR	QX-type	KX107789
CK/CH/YN/SL1301-1	Yunan	Jan.2013	broiler breeder	178	Egg production abnormal	1620nt/540aa	RRSRR	4/91-type	KX107836

CK/CH/YN/SL1301-2	Yunan	Jan.2013	broiler	36	Nephropathogenic	1620nt/540aa	RRSRR	4/91-type	KX107837
CK/CH/ZJ/NX1301	Zhejiang	Jan.2013	broiler	55	Nephropathogenic	1620nt/540aa	HRRRR	QX-type	KX107845
CK/CH/ZJ/QZ1301-2	Zhejiang	Jan.2013	broiler	21	Nephropathogenic	1617nt/539aa	HRRRR	QX-type	KX107847
CK/CH/ZJ/QZ1301-1	Zhejiang	Jan.2013	broiler	62	Nephropathogenic	1620nt/540aa	HRRRR	QX-type	KX107846
CK/CH/AH/CZ1302-3	Anhui	Feb.2013	broiler breeder	176	Egg production abnormal	1620nt/540aa	HRRRR	QX-type	KX107632
CK/CH/AH/CZ1302-4	Anhui	Feb.2013	broiler	40	Respiratory	1623nt/541aa	RRFRR	TW I-type	KX107633
CK/CH/AH/CZ1302-2	Anhui	Feb.2013	broiler	15	Nephropathogenic	1620nt/540aa	HRRRR	QX-type	KX107631
CK/CH/AH/CZ1302-1	Anhui	Feb.2013	broiler	40	Nephropathogenic	1620nt/540aa	HRRRR	QX-type	KX107630
CK/CH/AH/HF1302-1	Anhui	Feb.2013	broiler	19	Nephropathogenic	1620nt/540aa	HRRRR	QX-type	KX107635
CK/CH/AH/HF1302-2	Anhui	Feb.2013	broiler	24	Nephropathogenic	1620nt/540aa	HRRRR	QX-type	KX107636
CK/CH/AH/HF1303	Anhui	Mar.2013	broiler	26	Nephropathogenic	1620nt/540aa	HRRRR	QX-type	KX107637
CK/CH/GX/GL1303-3	Guangxi	Mar.2013	broiler	16	Nephropathogenic	1620nt/540aa	RRFRR	TW I-type	KX107698
CK/CH/GX/GL1303-1	Guangxi	Mar.2013	broiler	17	Nephropathogenic	1620nt/540aa	HRRRR	QX-type	KX107696
CK/CH/GX/GL1303-2	Guangxi	Mar.2013	broiler	37	Nephropathogenic	1620nt/540aa	HRSRR	4/91-type	KX107697
CK/CH/GX/GL1303-4	Guangxi	Mar.2013	broiler	58	Nephropathogenic	1620nt/540aa	RRFRR	TW I-type	KX107699
CK/CH/GX/NN1303	Guangxi	Mar.2013	broiler	23	Nephropathogenic	1620nt/540aa	HRRRR	QX-type	KX107721
CK/CH/HuB/HC1303-2	Hubei	Mar.2013	broiler	34	Respiratory	1617nt/539aa	HRRRR	4/91-type	KX107741
CK/CH/HuB/HC1303-4	Hubei	Mar.2013	broiler	13	Nephropathogenic	1617nt/539aa	HRRRR	4/91-type	KX107743
CK/CH/HuB/HC1303-3	Hubei	Mar.2013	broiler	22	Nephropathogenic	1617nt/539aa	HRRRR	4/91-type	KX107742
CK/CH/HuB/HC1303-1	Hubei	Mar.2013	broiler	38	Nephropathogenic	1617nt/539aa	HRRRR	4/91-type	KX107740
CK/CH/HuN/NX1303	Hunan	Mar.2013	broiler	22	Nephropathogenic	1620nt/540aa	HRRRR	QX-type	KX107797
CK/CH/AH/CZ1304	Anhui	Apr.2013	broiler	21	Nephropathogenic	1620nt/540aa	HRRRR	QX-type	KX107634
CK/CH/FJ/ZZ1304	Fujian	Apr.2013	broiler	22	Nephropathogenic	1620nt/540aa	RRFRR	TW I-type	KX107652
CK/CH/GX/NN1304	Guangxi	Apr.2013	broiler	6	Nephropathogenic	1620nt/540aa	HRRRR	QX-type	KX107722
CK/CH/HuB/HC1304-2	Hubei	Apr.2013	broiler breeder	155	Egg production abnormal	1617nt/539aa	HRSRR	4/91-type	KX107745
CK/CH/HuB/HC1304-1	Hubei	Apr.2013	broiler	53	Nephropathogenic	1617nt/539aa	HRRRR	4/91-type	KX107744

CK/CH/HuB/WH1304-2	Hubei	Apr.2013	broiler	17	Nephropathogenic	1620nt/540aa	HRRRR	QX-type	KX107780
CK/CH/HuB/WH1304-1	Hubei	Apr.2013	broiler	45	Nephropathogenic	1620nt/540aa	HRRRR	4/91-type	KX107779
CK/CH/HuN/NX1304-2	Hunan	Apr.2013	broiler	14	Nephropathogenic	1620nt/540aa	HRRRR	QX-type	KX107799
CK/CH/HuN/NX1304-1	Hunan	Apr.2013	broiler	44	Nephropathogenic	1620nt/540aa	HRRRR	QX-type	KX107798
CK/CH/GX/NN1306	Guangxi	Jun.2013	broiler	14	Nephropathogenic	1617nt/539aa	RRSRR	4/91-type	KX107723
CK/CH/GX/NN1307	Guangxi	Jul.2013	broiler	21	Nephropathogenic	1620nt/540aa	RRFRR	LDT3-type	KX107724
CK/CH/HuB/WH1307	Hubei	Jul.2013	broiler	60	Nephropathogenic	1620nt/540aa	HRRRR	QX-type	KX107781
CK/CH/GX/NN1308	Guangxi	Aug.2013	broiler	45	Nephropathogenic	1611nt/537aa	RRFRR	Mass-type	KX107725
CK/CH/GX/ZS13	Guangxi	Aug.2013	broiler	20	Nephropathogenic	1620nt/540aa	RRFRR	TW I-type	KX107733
CK/CH/AH/HF1309	Anhui	Sept.2013	broiler	18	Nephropathogenic	1620nt/540aa	HRRRR	QX-type	KX107638
CK/CH/FJ/FS1310-2	Fujian	Sept.2013	broiler	19	Nephropathogenic	1620nt/540aa	RRFRR	TW I-type	KX107644
CK/CH/FJ/FS1310-1	Fujian	Sept.2013	broiler	52	Nephropathogenic	1620nt/540aa	RRSRR	4/91-type	KX107643
CK/CH/FJ/FS1310-3	Fujian	Sept.2013	broiler	55	Nephropathogenic	1620nt/540aa	RRFRR	TW I-type	KX107645
CK/CH/HuB/WH1310	Hubei	Sept.2013	broiler	24	Nephropathogenic	1620nt/540aa	RRFRR	LDT3-type	KX107782
CK/CH/HuN/NX1310-1	Hunan	Sept.2013	broiler	5	Nephropathogenic	1620nt/540aa	HRRRR	QX-type	KX107800
CK/CH/HuN/NX1310-2	Hunan	Sept.2013	broiler	34	Nephropathogenic	1620nt/540aa	RRFRR	TW I-type	KX107801
CK/CH/CQ/1310-2	chongqing	Sept.2013	broiler	23	Nephropathogenic	1620nt/540aa	HRRRR	QX-type	KX107642
CK/CH/CQ/1310-1	chongqing	Sept.2013	broiler	40	Nephropathogenic	1620nt/540aa	HRRRR	QX-type	KX107641
CK/CH/AH/HF1311	Anhui	Nov.2013	broiler	20	Nephropathogenic	1620nt/540aa	HRRRR	QX-type	KX107639
CK/CH/FJ/PanT1311	Fujian	Nov.2013	broiler	21	Nephropathogenic	1620nt/540aa	RRFRR	LDT3-type	KX107648
CK/CH/FJ/PT1311-2	Fujian	Nov.2013	broiler	25	Nephropathogenic	1620nt/540aa	RRFRR	TW I-type	KX107651
CK/CH/FJ/PT1311-1	Fujian	Nov.2013	broiler	31	Nephropathogenic	1620nt/540aa	RRFRR	LDT3-type	KX107650
CK/CH/GD/LZ1311	Guangdong	Nov.2013	broiler	55	Nephropathogenic	1617nt/539aa	RRSRR	4/91-type	KX107666
CK/CH/GX/GL1311-3	Guangxi	Nov.2013	broiler	18	Nephropathogenic	1620nt/540aa	RRFRR	TW I-type	KX107702
CK/CH/GX/GL1311-4	Guangxi	Nov.2013	broiler	28	Nephropathogenic	1620nt/540aa	RRFRR	TW I-type	KX107703
CK/CH/GX/GL1311-1	Guangxi	Nov.2013	broiler	40	Nephropathogenic	1617nt/539aa	RRSRR	4/91-type	KX107700

CK/CH/GX/GL1311-5	Guangxi	Nov.2013	broiler	40	Nephropathogenic	1620nt/540aa	RRFRR	TW I-type	KX107704
CK/CH/GX/GL1311-2	Guangxi	Nov.2013	broiler	45	Nephropathogenic	1617nt/539aa	RRSRR	4/91-type	KX107701
CK/CH/GX/GL1311-6	Guangxi	Nov.2013	broiler	55	Nephropathogenic	1620nt/540aa	RRFRR	TW I-type	KX107705
CK/CH/GX/YL1311	Guangxi	Nov.2013	broiler	42	Nephropathogenic	1620nt/540aa	HRRRR	QX-type	KX107730
CK/CH/HuB/HC1311	Hubei	Nov.2013	broiler	20	Nephropathogenic	1620nt/540aa	RRFRR	TW I-type	KX107746
CK/CH/HuB/WH1311	Hubei	Nov.2013	broiler	28	Nephropathogenic	1620nt/540aa	RRFRR	TW I-type	KX107783
CK/CH/SC/DY1311-2	Sichuan	Nov.2013	broiler	34	Respiratory	1620nt/540aa	HRRRR	QX-type	KX107828
CK/CH/SC/DY1311-1	Sichuan	Nov.2013	broiler	38	Nephropathogenic	1620nt/540aa	HRRRR	QX-type	KX107827
CK/CH/FJ/FS1312	Fujian	Dec.2013	broiler	30	Nephropathogenic	1620nt/540aa	HRRRR	QX-type	KX107646
CK/CH/GX/GL1312-1	Guangxi	Dec.2013	broiler	30	Nephropathogenic	1620nt/540aa	HRRRR	QX-type	KX107706
CK/CH/GX/GL1312-4	Guangxi	Dec.2013	broiler	30	Nephropathogenic	1620nt/540aa	RRFRR	TW I-type	KX107709
CK/CH/GX/GL1312-3	Guangxi	Dec.2013	broiler	31	Nephropathogenic	1620nt/540aa	HRRRR	QX-type	KX107708
CK/CH/GX/GL1312-5	Guangxi	Dec.2013	broiler	50	Nephropathogenic	1620nt/540aa	RRFRR	TW I-type	KX107710
CK/CH/GX/GL1312-2	Guangxi	Dec.2013	broiler	60	Nephropathogenic	1620nt/540aa	HRRRR	QX-type	KX107707
CK/CH/HuB/HC1312-3	Hubei	Dec.2013	broiler breeder	178	Egg production abnormal	1617nt/539aa	HRRRR	QX-type	KX107749
CK/CH/HuB/HC1312-4	Hubei	Dec.2013	broiler	17	Nephropathogenic	1617nt/539aa	HRRRR	QX-type	KX107750
CK/CH/HuB/HC1312-1	Hubei	Dec.2013	broiler	32	Nephropathogenic	1620nt/540aa	HRRRR	QX-type	KX107747
CK/CH/HuB/HC1312-2	Hubei	Dec.2013	broiler	36	Nephropathogenic	1620nt/540aa	RRLRR	TW I-type	KX107748
CK/CH/HuB/JL1312	Hubei	Dec.2013	broiler	15	Nephropathogenic	1620nt/540aa	RRFRR	TW I-type	KX107768
CK/CH/HuB/WH1312-2	Hubei	Dec.2013	broiler	14	Respiratory	1617nt/539aa	HRRRR	QX-type	KX107785
CK/CH/HuB/WH1312-1	Hubei	Dec.2013	broiler	13	Nephropathogenic	1620nt/540aa	RRFRR	LDT3-type	KX107784
CK/CH/ZJ/HZ1312	Zhejiang	Dec.2013	broiler	19	Nephropathogenic	1620nt/540aa	HRRRR	QX-type	KX107842
CK/CH/AH/HF1401	Anhui	Jan.2014	broiler	28	Nephropathogenic	1620nt/540aa	RRFRR	LDT3-type	KX107640
CK/CH/GD/HY1401	Guangdong	Jan.2014	broiler	41	Nephropathogenic	1620nt/540aa	RRFRR	TW I-type	KX107661
CK/CH/GD/LZ1401-1	Guangdong	Jan.2014	broiler breeder	174	Egg production abnormal	1617nt/539aa	HRRRR	Variant	KX107667
CK/CH/GD/LZ1401-2	Guangdong	Jan.2014	broiler	29	Nephropathogenic	1620nt/540aa	HRRRR	QX-type	KX107668

CK/CH/GD/LZ1401-3	Guangdong	Jan.2014	broiler	36	Nephropathogenic	1626nt/542aa	HRRKR	QX-type	KX107669
CK/CH/GD/XX1401-3	Guangdong	Jan.2014	broiler	16	Nephropathogenic	1620nt/540aa	RRFRR	TW I-type	KX107678
CK/CH/GD/XX1401-2	Guangdong	Jan.2014	broiler	20	Nephropathogenic	1620nt/540aa	RRFRR	TW I-type	KX107677
CK/CH/GD/XX1401-1	Guangdong	Jan.2014	broiler	45	Nephropathogenic	1620nt/540aa	RRFRR	TW I-type	KX107676
CK/CH/HuB/HC1401-1	Hubei	Jan.2014	broiler	24	Nephropathogenic	1620nt/540aa	HRRKR	QX-type	KX107751
CK/CH/HuB/HC1401-2	Hubei	Jan.2014	broiler	28	Nephropathogenic	1620nt/540aa	RRFRR	TW I-type	KX107752
CK/CH/HuB/JL1401-1	Hubei	Jan.2014	broiler	22	Nephropathogenic	1620nt/540aa	HRRRR	QX-type	KX107769
CK/CH/HuB/JL1401-2	Hubei	Jan.2014	broiler	36	Nephropathogenic	1620nt/540aa	HRRRR	QX-type	KX107770
CK/CH/HuN/NTP1401-2	Hunan	Jan.2014	broiler	4	Nephropathogenic	1611nt/537aa	RRFRR	Mass-type	KX107793
CK/CH/HuN/NTP1401-1	Hunan	Jan.2014	broiler	12	Nephropathogenic	1620nt/540aa	RRFRR	TW I-type	KX107792
CK/CH/HuN/NX1401-3	Hunan	Jan.2014	broiler	18	Nephropathogenic	1620nt/540aa	RRFRR	TW I-type	KX107804
CK/CH/HuN/NX1401-1	Hunan	Jan.2014	broiler	23	Nephropathogenic	1620nt/540aa	HRRRR	QX-type	KX107802
CK/CH/HuN/NX1401-2	Hunan	Jan.2014	broiler	38	Nephropathogenic	1620nt/540aa	RRFRR	TW I-type	KX107803
CK/CH/SC/MS1401	Sichuan	Jan.2014	broiler	32	Nephropathogenic	1620nt/540aa	RRFRR	TW I-type	KX107829
CK/CH/HuB/HC1402-2	Hubei	Feb.2014	broiler	14	Nephropathogenic	1620nt/540aa	HRRRR	QX-type	KX107754
CK/CH/HuB/HC1402-4	Hubei	Feb.2014	broiler	15	Nephropathogenic	1620nt/540aa	RRFRR	TW I-type	KX107756
CK/CH/HuB/HC1402-3	Hubei	Feb.2014	broiler breeder	160	Nephropathogenic	1617nt/539aa	HRRRR	4/91-type	KX107755
CK/CH/HuB/JL1402-1	Hubei	Feb.2014	broiler	25	Respiratory	1620nt/540aa	HRRRR	QX-type	KX107771
CK/CH/HuB/JL1402-3	Hubei	Feb.2014	broiler	13	Nephropathogenic	1620nt/540aa	HRRRR	QX-type	KX107773
CK/CH/HuB/JL1402-2	Hubei	Feb.2014	broiler	17	Nephropathogenic	1620nt/540aa	HRRRR	QX-type	KX107772
CK/CH/HuB/WH1401	Hubei	Feb.2014	broiler	27	Nephropathogenic	1617nt/539aa	HRRRR	QX-type	KX107786
CK/CH/HuB/XN1402	Hubei	Feb.2014	broiler	16	Nephropathogenic	1620nt/540aa	HRRRR	QX-type	KX107791
CK/CH/JS/ZJ1402-2	Jiangsu	Feb.2014	broiler	6	Nephropathogenic	1617nt/539aa	HRRRR	QX-type	KX107823
CK/CH/JS/ZJ1402-1	Jiangsu	Feb.2014	broiler	18	Nephropathogenic	1617nt/539aa	HRRRR	QX-type	KX107822
CK/CH/JS/ZJ1402-3	Jiangsu	Feb.2014	broiler	35	Nephropathogenic	1620nt/540aa	RRFRR	TW I-type	KX107824
CK/CH/HuB/HC1403	Hubei	Mar.2014	broiler	14	Nephropathogenic	1620nt/540aa	RRFRR	TW I-type	KX107757

CK/CH/GX/GL1404	Guangxi	Apr.2014	broiler	27	Nephropathogenic	1635nt/545aa	HRHKR	CH VI-type	KX107711
CK/CH/JS/ZJ1404	Jiangsu	Apr.2014	broiler	23	Nephropathogenic	1620nt/540aa	HRRRR	QX-type	KX107825
CK/CH/GD/GZ14	Guangdong	May.2014	broiler	22	Nephropathogenic	1620nt/540aa	RRFRR	TW I-type	KX107660
CK/CH/YN/SL1405-2	Yunan	May.2014	broiler breeder	154	Egg production abnormal	1620nt/540aa	HRRRR	QX-type	KX107839
CK/CH/YN/SL1405-3	Yunan	May.2014	broiler	38	Nephropathogenic	1620nt/540aa	RRFRR	TW I-type	KX107840
CK/CH/YN/SL1405-1	Yunan	May.2014	broiler	55	Nephropathogenic	1620nt/540aa	HRRRR	QX-type	KX107838
CK/CH/FJ/ZZ1406-1	Fujian	Jun.2014	broiler breeder	166	Egg production abnormal	1620nt/540aa	RRSRR	4/91-type	KX107653
CK/CH/FJ/ZZ1406-2	Fujian	Jun.2014	broiler breeder	176	Egg production abnormal	1620nt/540aa	RRFRR	4/91-type	KX107654
CK/CH/HuN/NTP1406-2	Hunan	Jun.2014	broiler	24	Nephropathogenic	1620nt/540aa	HRRRR	QX-type	KX107795
CK/CH/HuN/NTP1406-1	Hunan	Jun.2014	broiler breeder	119	Nephropathogenic	1620nt/540aa	HRRRR	QX-type	KX107794
CK/CH/HuN/NX1406	Hunan	Jun.2014	broiler	19	Nephropathogenic	1617nt/539aa	HRRRR	QX-type	KX107805
CK/CH/ZJ/HZ1406-2	Zhejiang	Jun.2014	broiler	31	Nephropathogenic	1620nt/540aa	HRRRR	QX-type	KX107844
CK/CH/ZJ/HZ1406-1	Zhejiang	Jun.2014	broiler	41	Nephropathogenic	1620nt/540aa	HRRRR	QX-type	KX107843
CK/CH/SD/TA1407	shandong	Jul.2014	broiler	40	Nephropathogenic	1620nt/540aa	HRRRR	QX-type	KX107830
CK/CH/HuB/HC1408-2	Hubei	Aug.2014	broiler	20	Respiratory	1617nt/539aa	RRSRR	4/91-type	KX107759
CK/CH/HuB/HC1408-1	Hubei	Aug.2014	broiler	27	Nephropathogenic	1617nt/539aa	HRRRR	QX-type	KX107758
CK/CH/SD/TA1409	shandong	Sept.2014	broiler	46	Nephropathogenic	1620nt/540aa	RRFRR	LDT3-type	KX107831
CK/CH/FJ/FS1410	Fujian	Oct.2014	broiler	23	Nephropathogenic	1620nt/540aa	RRSRR	4/91-type	KX107647
CK/CH/GD/HY1410	Guangdong	Oct.2014	broiler breeder	170	Egg production abnormal	1620nt/540aa	RRSRR	4/91-type	KX107662
CK/CH/GD/XX1410	Guangdong	Oct.2014	broiler	38	Nephropathogenic	1620nt/540aa	RRLRR	TW I-type	KX107679
CK/CH/GD/HY1411	Guangdong	Nov.2014	broiler	30	Nephropathogenic	1620nt/540aa	RRFRR	TW I-type	KX107663
CK/CH/GD/RC1411	Guangdong	Nov.2014	broiler	20	Nephropathogenic	1620nt/540aa	RRFRR	TW I-type	KX107673
CK/CH/GD/XX1411-1	Guangdong	Nov.2014	broiler	12	Nephropathogenic	1620nt/540aa	RRSRR	4/91-type	KX107680
CK/CH/GD/XX1411-2	Guangdong	Nov.2014	broiler	38	Nephropathogenic	1620nt/540aa	RRFRR	TW I-type	KX107681
CK/CH/JS/TC1411-1	Jiangsu	Nov.2014	broiler	16	Nephropathogenic	1620nt/540aa	HRRRR	QX-type	KX107817
CK/CH/JS/TC1411-3	Jiangsu	Nov.2014	broiler	17	Nephropathogenic	1620nt/540aa	HRRRR	QX-type	KX107819

CK/CH/JS/TC1411-5	Jiangsu	Nov.2014	broiler	23	Nephropathogenic	1620nt/540aa	HRRRR	QX-type	KX107821
CK/CH/JS/TC1411-2	Jiangsu	Nov.2014	broiler	31	Nephropathogenic	1620nt/540aa	HRRRR	QX-type	KX107818
CK/CH/SD/TA1411-2	shandong	Nov.2014	broiler	22	Respiratory	1620nt/540aa	HRRRR	QX-type	KX107833
CK/CH/SD/TA1411-1	shandong	Nov.2014	broiler	23	Nephropathogenic	1620nt/540aa	HRRRR	QX-type	KX107832
CK/CH/GD/CG1412-2	Guangdong	Dec.2014	broiler	14	Respiratory	1620nt/540aa	RRFRR	TW I-type	KX107658
CK/CH/GD/CG1412-1	Guangdong	Dec.2014	broiler	10	Nephropathogenic	1620nt/540aa	RRFRR	TW I-type	KX107657
CK/CH/GD/CG1412-3	Guangdong	Dec.2014	broiler	20	Nephropathogenic	1611nt/537aa	RRFRR	Mass-type	KX107659
CK/CH/GD/HY1412	Guangdong	Dec.2014	broiler	35	Nephropathogenic	1620nt/540aa	RRFRR	TW I-type	KX107664
CK/CH/GD/LZ1412	Guangdong	Dec.2014	broiler	31	Nephropathogenic	1620nt/540aa	RRFRR	TW I-type	KX107671
CK/CH/GD/LD1412	Guangdong	Dec.2014	broiler	52	Nephropathogenic	1620nt/540aa	HRRRR	QX-type	KX107665
CK/CH/GD/XX1412-6	Guangdong	Dec.2014	broiler breeder	172	Egg production abnormal	1620nt/540aa	RRFRR	TW I-type	KX107687
CK/CH/GD/XX1412-1	Guangdong	Dec.2014	broiler	14	Nephropathogenic	1620nt/540aa	RRFRR	LDT3-type	KX107682
CK/CH/GD/XX1412-4	Guangdong	Dec.2014	broiler	19	Nephropathogenic	1620nt/540aa	RRSRR	4/91-type	KX107685
CK/CH/GD/XX1412-3	Guangdong	Dec.2014	broiler	23	Nephropathogenic	1620nt/540aa	RRFRR	TW I-type	KX107684
CK/CH/GD/XX1412-5	Guangdong	Dec.2014	broiler	23	Nephropathogenic	1620nt/540aa	RRSRR	4/91-type	KX107686
CK/CH/GD/XX1412-2	Guangdong	Dec.2014	broiler	30	Nephropathogenic	1635nt/545aa	HRHKR	CH VI-type	KX107683
CK/CH/GX/GL1412-1	Guangxi	Dec.2014	broiler	14	Nephropathogenic	1620nt/540aa	RRFRR	TW I-type	KX107712
CK/CH/GX/GL1412-2	Guangxi	Dec.2014	broiler	17	Nephropathogenic	1620nt/540aa	RRFRR	TW I-type	KX107713
CK/CH/GX/ZS1412	Guangxi	Dec.2014	broiler	5	Nephropathogenic	1620nt/540aa	RRSRR	4/91-type	KX107738
CK/CH/GD/XX1501-4	Guangdong	Jan.2015	broiler breeder	189	Egg production abnormal	1620nt/540aa	RRFRR	TW I-type	KX107691
CK/CH/GD/XX1501-3	Guangdong	Jan.2015	broiler	17	Nephropathogenic	1620nt/540aa	RRFRR	TW I-type	KX107690
CK/CH/GD/XX1501-1	Guangdong	Jan.2015	broiler	18	Nephropathogenic	1620nt/540aa	HRRRR	QX-type	KX107688
CK/CH/GD/XX1501-2	Guangdong	Jan.2015	broiler	24	Nephropathogenic	1620nt/540aa	RRSRR	4/91-type	KX107689
CK/CH/GX/YL1501-2	Guangxi	Jan.2015	broiler	19	Nephropathogenic	1635nt/545aa	HRHKR	CH VI-type	KX107732
CK/CH/GX/YL1501-1	Guangxi	Jan.2015	broiler	30	Nephropathogenic	1620nt/540aa	RRFRR	TW I-type	KX107731
CK/CH/GX/ZS1501	Guangxi	Jan.2015	broiler	23	Nephropathogenic	1620nt/540aa	RRFRR	TW I-type	KX107739

CK/CH/HuB/HC1501	Hubei	Jan.2015	broiler	23	Respiratory	1617nt/539aa	HRRRR	QX-type	KX107760
CK/CH/HuB/WH1501	Hubei	Jan.2015	broiler	20	Respiratory	1617nt/539aa	HRRRR	QX-type	KX107787
CK/CH/YN/SL1501	Yunan	Jan.2015	broiler	8	Nephropathogenic	1617nt/539aa	RRSRR	4/91-type	KX107841
CK/CH/JS/HA1502-1	Jiangsu	Feb.2015	broiler breeder	245	Egg production abnormal	1620nt/540aa	HRRRR	QX-type	KX107806
CK/CH/JS/HA1502-2	Jiangsu	Feb.2015	broiler	19	Respiratory	1620nt/540aa	HRRRR	QX-type	KX107807
CK/CH/JS/LYG1502	Jiangsu	Feb.2015	broiler	45	Nephropathogenic	1620nt/540aa	HRRRR	QX-type	KX107814
CK/CH/JS/ZJ1502	Jiangsu	Feb.2015	broiler	30	Nephropathogenic	1614nt/538aa	RRFRR	4/91-type	KX107826
CK/CH/SD/TA1502-1	shandong	Feb.2015	broiler	21	Nephropathogenic	1620nt/540aa	HRRRR	QX-type	KX107834
CK/CH/SD/TA1502-2	shandong	Feb.2015	broiler	26	Nephropathogenic	1620nt/540aa	HRRRR	TW I-type	KX107835
CK/CH/JS/HA1504-2	Jiangsu	Mar.2015	broiler	10	Nephropathogenic	1611nt/537aa	RRFRR	Mass-type	KX107809
CK/CH/JS/HA1504-1	Jiangsu	Mar.2015	broiler	49	Nephropathogenic	1620nt/540aa	HRRRR	QX-type	KX107808
CK/CH/JS/HA1505-2	Jiangsu	May.2015	broiler	20	Nephropathogenic	1620nt/540aa	HRRRR	QX-type	KX107811
CK/CH/JS/HA1505-1	Jiangsu	May.2015	broiler	22	Nephropathogenic	1611nt/537aa	RRFRR	Mass-type	KX107810
CK/CH/JS/LYG15	Jiangsu	May.2015	broiler	16	Nephropathogenic	1620nt/540aa	HRRRR	TW I-type	KX107813
CK/CH/JS/HA1506	Jiangsu	Jue.2015	broiler	36	Nephropathogenic	1620nt/540aa	HRRRR	QX-type	KX107812
CK/CH/JS/LYG1506-1	Jiangsu	Jue.2015	broiler	47	Nephropathogenic	1620nt/540aa	HRRRR	TW I-type	KX107815
CK/CH/GX/GL15	Guangxi	Jul.2015	broiler breeder	184	Egg production abnormal	1620nt/540aa	HRRRR	QX-type	KX107714
CK/CH/FJ/ZZ1508	Fujian	Aug.2015	broiler	30	Respiratory	1620nt/540aa	HRRRR	QX-type	KX107656
CK/CH/GD/LZ15	Guangdong	Aug.2015	broiler breeder	180	Egg production abnormal	1620nt/540aa	RRFRR	TW I-type	KX107672
CK/CH/GX/GL1511	Guangxi	Nov.2015	broiler	31	Nephropathogenic	1635nt/545aa	HRHKR	CH VI-type	KX107715
CK/CH/HuB/HC1511-2	Hubei	Nov.2015	broiler	18	Respiratory	1620nt/540aa	RRFRR	LDT3-type	KX107762
CK/CH/GX/NN1512	Guangxi	Dec.2015	broiler breeder	72	Nephropathogenic	1611nt/537aa	RRFRR	Mass-type	KX107727
CK/CH/HuB/HC1512-3	Hubei	Dec.2015	broiler	17	Nephropathogenic	1620nt/540aa	HRRRR	QX-type	KX107765
CK/CH/HuB/HC1512-1	Hubei	Dec.2015	broiler	22	Nephropathogenic	1620nt/540aa	HRRRR	QX-type	KX107763
CK/CH/HuB/HC1512-4	Hubei	Dec.2015	broiler	25	Nephropathogenic	1620nt/540aa	HRRRR	QX-type	KX107766
CK/CH/HuB/HC1512-2	Hubei	Dec.2015	broiler	35	Nephropathogenic	1620nt/540aa	HRRRR	QX-type	KX107764

CK/CH/HuB/HC1512-5	Hubei	Dec.2015	broiler	38	Nephropathogenic	1617nt/539aa	HRRRR	QX-type	KX107767
CK/CH/HuB/JL1512-1	Hubei	Dec.2015	broiler	38	Respiratory	1620nt/540aa	HRRRR	QX-type	KX107775
CK/CH/HuB/JL1512-2	Hubei	Dec.2015	broiler	30	Nephropathogenic	1620nt/540aa	HRRRR	QX-type	KX107776
CK/CH/HuB/WH1512	Hubei	Dec.2015	broiler	23	Respiratory	1620nt/540aa	HRRRR	QX-type	KX107788

[a] Province where the virus were isolated.

[b] Length of nucleotide and deduced amino acids of S1 glycoprotein gene

[c] Cleavage recognition motifs of S1 glycoprotein gene

[d] R arginine, F phenylalanine, H histidine, T threonine, K Lysine, L Leucine

Supplement Table S2 Universally nucleotides variations between Cluster I and Cluster II QX-type strains

Position (nt)[a]	Nucleotide of QXIBV	Nucleotide of Cluster I strains and proportion[b]	Nucleotide of Cluster II strains and proportion[c]
427	T	T 46/48, A 2/48	T 14/47, A 33/47
477	A	G 40/48, A 8/48	G 11/47, A 36/47
489	T	T 48/48	T 11/47, C 36/47
470	A	C 8/48, G 40/48	G 6/47, A 41/47
514	T	C 7/48, T 41/48	T 9/47, C 38/47
489	T	T 48/48	T 11/47, C 36/47
507	G	G 48/48	G 8/47, A 39/47
752	G	G 48/48	G 9/47, C 38/47
768	A	C 48/48	C 1/47, A 46/47
777	T	T 48/48	T 4/47, C 43/47
786	C	T 48/48	T 4/47, C 43/47
808	C	T 48/48	T 1/47, C 46/47
811	G	G 47/48, A 1/48	G 46/47, A 1/47
872	A	G 47/48, A 1/48	G 1/47, A 46/47
1029	G	G 48/48	G 46/47, A 1/47
1041	C	C 46/48, T 2/48	C 10/47, T 37/47
1056, 1057	TA	TA 48/48	TA 1/48, AG 47/48
1068	C	C 48/48	A 46/47, C 1/47
1074	G	G 48/48	A 47/47
1089	A	A 48/48	G 47/47
1119	T	T 48/48	T 3/47, C 44/47
1124-1147	GGG	GGG 48/48	GGG 1/47, TAG 46/47
1170	T	T 48/48	T 1/47, C 46/47
1177-1182	AGCATG	AGCCAG 6/48, AGCAAG 42/48	CGGACT 23/47, CAGACT 22/47, AAGCAA, 2/47
1204	G	G 47/48, A 1/48	A 47/47
1248	G	A 3/48, G 45/48	A 47/47
1265	C	C 4/48, T 44/48	C 46/47, T 1/47

a The nucleotide positions correspond to S1 gene sequence of QXIBV, GenBank Accession number KC795604.

b 48 strains of Cluster I QX-type IBV were in the sequence alignment.

c 47 strains of Cluster II QX-type IBV were in the sequence alignment.

Supplement Table S3 IBV reference strains published in GenBank

IBV strain	Year of isolation	geographic origin	Serotype/Genotype/ Pathogenicity type	S1 cleavage recognition motifs	Accession number
Ma5	N/A[a]	Vaccine strain	Mass serotype	RRFRR[b]	AY561713
H120	N/A	Vaccine strain	Mass serotype	RRFRR	EU822341
H52	N/A	Vaccine strain	Mass serotype	RRFRR	AF352315
W93	N/A	Vaccine strain	Mass serotype	RRFRR	AY846750
4/91 vaccine	N/A	Vaccine strain	793/B serotype	RRSRR	KF377577
M41	1956	USA	Mass serotype	RRFRR	DQ834384
ZJ971	1997	Zhejiang, China	Preventriculus	RRFRR	AF352313
Beaudette	1937	USA	Mass serotype	RRFRR	M95196
Ark99	1973	USA	Arkansas serotype	HRSRR	M99482
Gray	1962	USA	Gray serotype	RRSRR	L14069
Holte	1962	USA	Holte serotype	RRSRR	L18988
7/93	1993	UK	793/B serotype	RRSRR	Z83979
4/91 pathogenic	1991	UK	793/B serotype	RRSRR	AF093794
TW2575/98	1998	Taiwan	TW I	RRFRR	DQ646405
TW3468/07	2007	Taiwan	TW I	RRSRR	EU822336
TW3071/3	2003	Taiwan	TW I	RRFRR	AY606319
TW3263/04	2004	Taiwan	TW II	RRFRR	EU822338
T07/02	2007	Taiwan	TW II	RRFRR	AY606322
LX4	1999	Xinjiang, China	Nephropathogenic	HRRRR	AY338732
QXIBV	1997	Shandong, China	Preventriculus	HRRRR	AF193423
A2	1996	China	Nephropathogenic	HRRRR	AY043312
DE072	1972	USA	Nephropathogenic	RRIRR	AF274435
LDT3A	2003	Vaccine strain	LDT3 genotype	RRFRR	KR608272
BJ	N/A	Beijing, China	Nephropathogenic	RRTRR	AY319651
CK/CH/LDL/97I	1997	Liaoning, China	Preventriculus	RRTGR	JX195178
Australian T	N/A	Australian	Nephropathogenic	RRSRR	AY775779
JP9758	1997	Japan	N/A	RRFKR	AY296746
YX10	2010	Zhejiang, China	Nephropathogenic	HRRRR	JX840411
CK/CH/LSC/99I	1999	Sichuan, China	Preventriculus	RRFRR	DQ167147
CK/CH/LHLJ/95I	1995	Heilongjiang, China	Nephropathogenic	HRRRR	DQ167141
DY05	2005	Sichuan, China	Nephropathogenic	HRRRR	GQ265928
PSH050513	2005	Guangdong, China	Nephropathogenic	RRFRR	DQ160004
N1-62	1962	Australian	Nephropathogenic	RRSRR	DQ490206
HN08	2008	Henan, China	Nephropathogenic	RRFRR	GQ265940
SAIBK	2007	Sichuan, China	Nephropathogenic	RRFRR	DQ288927
TC07-2	2007	Jiangsu, China	Respiratory	HRRKR	GQ265948
TA03	2003	Shandong, China	793/B serotype	RRSRR	AY837465
CK/CH/GD/KP10	2010	Guangdong, China	Respiratory	HRHKR	HQ018919
CK/CH/GD/NC10	2010	Guangdong, China	Nephropathogenic	HRHKR	HQ018903
CK/CH/SC/ZJ10-1	2010	Sichuan, China	Nephropathogenic	RRFRR	HQ018918

CK/CH/HuN/NX09	2009	Hunan, China	Nephropathogenic	RRFRR	HQ018899
CK/CH/GX/YL09-2	2009	Guangxi, China	Nephropathogenic	HRRRR	HQ018905

a N/A date not available

b R arginine, F phenylalanine, H histidine, S serine, T threonine, I Isoleucine, G Glycine, K, Lysine
